# Nurses’ attitudes and stress related to perinatal bereavement care in Korea: a cross-sectional survey

**DOI:** 10.4069/kjwhn.2022.05.24.1

**Published:** 2022-06-17

**Authors:** Eunhui Kim, Hae Won Kim

**Affiliations:** 1 Department of Nursing, Seoul National University Bundang Hospital, Seongnam, Korea; 2 College of Nursing and The Research Institute of Nursing Science, Center for Human-Caring Nurse Leaders for the Future by Brain Korea 21 (BK 21) Four Project, Seoul National University, Seoul, Korea

**Keywords:** Attitude, Bereavement, Perinatal death

## Abstract

**Purpose:**

A descriptive correlational survey was conducted to examine nurses’ attitudes and stress related to perinatal bereavement care (PBC) and their relationships, with the ultimate goal of improving nurses’ capabilities related to PBC.

**Methods:**

Korean nurses (N=136) who had experienced perinatal death at least once were recruited from seven hospitals. Data were analyzed using descriptive statistics. The Korean version of Nurses’ Attitudes towards Perinatal Bereavement Support was assessed in terms of three subdomains (attitudes to PBC, importance of policies related to PBC, and importance of training related to PBC), and nurses’ stress was measured.

**Results:**

The participants gave high scores for the attitude-related items of “giving sufficient time to bereaved parents to mourn for their dead baby” (4.54 points) and “nurses should treat bereaved parents with respect and dignity” (4.51 points), and they perceived a high level of importance for the policy-related items of “every staff member in the hospital should understand the policies relevant for PBC” and “when nurses feel emotional exhaustion, they should seek support” (4.58 points). Nurses’ attitude toward PBC was associated with the perceived importance of policies (r=.40, *p*<.001), the perceived importance of PBC-related training (r=.61, *p*<.001), and stress related to PBC (r=.29, *p*<.001). Nurses’ perceived importance of PBC-related training was associated with stress related to PBC (r=.38, *p*<.001).

**Conclusion:**

Establishing hospital policies related to PBC and providing PBC training for nurses could positively affect nurses’ attitudes toward PBC. A stress management program for nurses could reduce the stress caused by PBC.

## Introduction

The perinatal mortality rate, which is an index that reflects the public health and health status of mothers and newborns [1], was 3.5 per 1,000 total births in 2009 (total number, 1,546) and 2.8 per 1,000 total births in 2018 in South Korea (total number, 904), but the rate increases as mothers become (hereafter, Korea) older [2]. Although several definitions are used for perinatal death based on the time of fetal death and postnatal death, the most common definition includes deaths at more than 20 weeks of gestation and death within 28 days of birth [3].

Perinatal death usually occurs suddenly, leading to feelings of guilt in women experiencing bereavement [4]; at the same time, nurses may experience feelings of failure and helplessness when caring for bereaved parents [5]. Perinatal bereavement care (PBC) refers to the comprehensive and integrative components of care provided by nurses and multidisciplinary staff, in the physical, psychological, emotional, and spiritual domains, following perinatal loss [6]. Recent guidelines have been proposed for nurses and other professionals to meet the needs of bereaved parents, recommending good communication, shared decision-making, recognition of parenthood, effective support, and organizational responses to enable the provision of high-quality PBC [7]. Nurses are the closest caregivers to parents who experience perinatal death and are perceived as the most helpful medical staff [8]. Nurses’ perceptions of caring activities related to perinatal death are related to their attitudes and education, as well as institutional policies [9]. That is, if a nurse has negative attitudes (e.g., fear and frustration) when treating a family who has suffered the death of a baby, it can result in a negative attitude toward the performance of care [10]. In contrast, knowledge and expertise with interventions related to parental mourning counseling can improve empathy and comprehension, helping to foster a positive nursing attitude that is helpful when performing actual nursing activities [9]. Nurses are also influenced by the existence of policies and protocols related to death and nursing in the workplace [11]. Maintaining high-quality policies could help to build a mourning culture to support parents’ and family members’ experiences of bereavement [12]. In addition, when a baby dies in the hospital, nurses experience extreme stress when providing support for the parents or family [13-15]. Nurses feel guilty under these circumstances [16], and persistent stress in nurses associated with perinatal death could negatively affect their perceptions of caring for patients affected perinatal death [17].

Several studies on nurses’ attitudes, perceptions, and stress related to PBC have been conducted in Israel, Hong Kong, and across the world [14,18-20], but it would be difficult to understand Korean nurses’ attitudes toward PBC and stress based on these findings because of differences in culture and health care systems. Therefore, this study examined Korean nurses’ attitudes and stress related to PBC, as well as their associations.

## Methods

Ethics statement: This study was approved by the Institutional Review Board of Seoul National University Bundang Hospital (B-1908-561-305). Informed consent was obtained from the participants.

### Study design

This descriptive correlational research was conducted to identify Korean nurses’ attitudes and stress related to PBC and to examine their relationships. The description followed the STROBE (Strengthening the Reporting of Observational Studies in Epidemiology) reporting guidelines (https://www.strobe-statement.org/).

### Setting and participants

The participants of this study were nurses working at the seven general hospitals located in Seoul and Gyeonggi Province, Korea, in departments that deal with perinatal death, i.e., labor and delivery, maternity unit, newborn nursery, and neonatal intensive care units (NICUs). This choice was made because the hospitals in Korea that provide PBC are mainly located in these regions. Participants were recruited through convenience sampling by the snowball method. The inclusion criteria were as follows: nurses who had been working in the maternity and neonatal related units for at least 1 year at the time of the survey and who had ever experienced at least one case of perinatal death. Nurses with less than 1 year of experience were excluded because perinatal bereavement was expected to be an infrequent experience.

### Sample size

The sample size was calculated using G*Power version 3.10, with a significance level of 0.05, power of 0.90, and a moderate effect size of 0.3. The required number of samples was determined to be 109. The questionnaire was distributed to a total of 150 nurses, considering a possible dropout rate of 20%. Out of the 148 participants who participated in the study, 12 incomplete responses were excluded and 136 questionnaires were ultimately analyzed.

### Measurements

#### Attitudes toward perinatal bereavement support

The Nurses’ Attitudes towards Perinatal Bereavement Support (NAPBS) scale [9] was used to measure nurses’ attitudes toward PBC and to identify required support and training needs for nurses on bereavement care. It consists of three subdomains: attitudes toward PBC (13 items), importance of policies related to PBC (four items), and importance of training related to PBC (eight items).

First, permission was obtained from Moon-Fai Chan, the developer of the NAPBS [9]. The original tool was first translated into Korean and reverse-translated into English. The translation did not focus on translating individual words and their meanings into Korean; instead, the core concepts were emphasized [21], with the goal that the end product would not feel like a translated tool. A written translation was again conducted (reverse translation) into English by the nurse. The three experts who participated in the first translation reviewed the equivalence between the reverse translation and the original version to finalize the translation. Then, the content validity of the translated tool was examined by 10 nursing experts. All 25 items had a content validity index of 0.8 or higher and were selected for inclusion in the final version. Prior to the study, the translated Korean version of the NAPBS was preliminarily tested among 10 nurses. Thereafter, the Korean version of the tool was finalized.

The final 25 questions are scored on a 5-point Likert score (1, ‘I do not agree at all’ to 5, ‘I strongly agree’). Higher summed scores for each subdomain (possible range: 13–65 for attitude, 4–20 for policy importance, 8–40 for training importance) correspond to more positive attitudes toward PBC or a greater recognition of the importance of policies or training related to PBC. Chan et al. [9] reported that Cronbach’s α was .92 for the total items, and .86, .83, .90 for the three subdomains of attitude, importance of policies, and importance of training, respectively. In the current study, Cronbach’s α was .87 for the total items, and .73, .67, and .90 for the three subdomains, respectively.

#### Stress related to perinatal bereavement care

The tool developed by Jang [22] was used in this study after receiving permission. This tool is composed of 29 questions in four domains: difficulties in providing care for patients affected by perinatal death (6 items), lack of knowledge (five items), inadequacies of the environment and systems for handling perinatal death (10 items), and psychological difficulties (eight items). Using a 5-point Likert score (1, ‘do not agree at all’ to 5, ‘strongly agree’), higher summed scores (possible range, 29–145) indicate high levels of stress. For all items, Jang [22] reported that Cronbach’s α was .87, while in the current study, Cronbach’s α was .89.

#### General characteristics

A questionnaire for general characteristics was developed from the literature. Information was gathered on participants’ demographic characteristics, including level of education, religion, and marital status. Additionally, information was collected on workplace, total career experience and experience at their current workplace, number of times PBC was experienced over the years, personal bereavement experience within the past year, they were also asked about whether they had ever received training on PBC. These factors were hypothesized to be associated with nurses’ perceptions and stress related to PBC based on the research framework [9].

### Data collection

Data were collected from September 1 to September 31, 2019, after obtaining permission from officials at the seven general hospitals in Seoul and Gyeonggi-do. Nurses working in the obstetrics or neonatal units received an explanation of the study purpose and procedures with a written protocol, and the questionnaire was distributed to nurses who voluntarily chose to participate. The researcher distributed questionnaires in an envelope individually and collected them in the same way to ensure confidentiality of the data. The participants received a small gift as a reward for participation (approximately 3 US dollars).

### Data analysis

Data were analyzed using IBM SPSS for Windows ver. 25.0 (IBM Corp., Armonk, NY, USA).

General characteristics and the main variables of the study were analyzed using descriptive statistics (mean, standard deviation, frequency, and percentage). NAPBS (attitude, importance of policies, and importance of training related to PBC) and PBC-related stress according to their general characteristics were analyzed using the independent t-test and one-way analysis of variance. Pearson correlation coefficients were calculated for continuous variables. The relationships among the three subdomains of NAPBS and stress were analyzed by Pearson correlation coefficients. All tests used a significance level of 0.05.

## Results

### General characteristics of the participants

The average age of the nurses was 31.33±6.76 years (range, 23–55 years), with the age group of 25 to 29 years comprising 44.9% of all participants. All of the participants were women, 48 (35.3%) were married (among whom 27 responded that they had children), and 79 (58.1%) had no religion. The majority (n=105, 77.2%) had a bachelor’s degree, and 19 (14.0%) stated that they had a master’s degree or higher.

Recent experiences (within the past year) of bereavement of a close friend or family member were reported by 22 of participants (16.2%). Only 10 (7.4%) had ever received education on bereavement care, and 69 (50.7%) had no policy related to bereavement care in their current workplace.

Overall, 82.4% of participants were staff nurses, 47.1% worked in the delivery room, and 44.1% worked in the NICU. On average, nurses’ clinical career duration was 100.17±81.93 months (range, 12–396 months) and 68 (50.0%) had at least 73 months of career experience. In terms of the total number of instances of PBC experienced by participants, 45 (33.1%) reported having experienced fewer than five instances, 43 (31.6%) reported having experienced 15 or more instances, and 28 participants (20.6%) reported having experienced PBC 5 to 9 times (Table 1).

### Nurses’ Attitudes towards Perinatal Bereavement Support and stress related to perinatal bereavement care

The average scores for the three subdomains of NAPBS were all at greater than midpoint level; 52.59±4.94 points for attitude toward PBC, 15.86±2.03 points for the importance of policies related to PBC, and 34.72±3.70 points for the importance of training on PBC. The item average scores for the subdomains were 4.05±0.38, 3.97±0.51, and 4.34±0.46, respectively, also at greater than midpoint level. Specifically, the highest scores were reported for “It is important to find support when feeling emotional exhaustion” (4.58 points), “We need to provide enough time for bereaved parents to mourn” (4.54 points), and “I will treat bereaved parents with respect and dignity” (4.51 points) (Table 2).

Regarding the nurses’ stress, the overall average score was greater than midpoint level (112.16 ± 13.46). The items with the highest scores were “caring for a dying newborn along with a heavy workload” (4.25 points) and “telling parents that their neonate has a poor prognosis” (4.25 points) (Table 3).

### Nurses’ Attitudes towards Perinatal Bereavement Support and stress related to perinatal bereavement care by general characteristics

Attitude toward PBC showed significant associations with having received training on PBC (t=–3.38, *p*=.001) and length of career experience in maternity and neonatal units (r=.22, *p*=.005). The importance of policies related to PBC showed significant associations with the existence of a policy in the workplace (t=–.423, *p*<.001), the number of experiences of PBC (F=4.47, *p*=.005), and length of career experience in related units (r=.19, *p*=.015). The importance of training for PBC was associated with length of career experience in related units (r=.16, *p*=.029), and the number of experiences of PBC (F=4.15, *p*=.008). However, no factor showed a significant relationship with nurses’ PBC-related stress (Table 4).

### Relationships among Nurses’ Attitudes towards Perinatal Bereavement Support and stress related to perinatal bereavement care

Nurses’ stress related to PBC was weakly correlated with their attitude toward PBC (r=.29, *p*<.001) and their perceived importance of training on PBC (r=.38, *p*<.001) (Table 5).

## Discussion

The overwhelming majority of the participants in this study (91.2%) had not received training on bereavement care, and nurses who had received education on PBC showed more positive attitudes toward PBC than those who had not. Participants’ responses revealed a high degree of necessity of training for PBC, and participants simultaneously reported a high level of stress due to a knowledge deficit when providing nursing care for a dying baby. PBC education for health care professionals has been shown to be effective for enhancing their perceptions of emotional support for bereaved parents [23]. Bereavement services for nurses can be improved by including guidelines, policies, and educational support, the most important components of which are education and training on adequate communication with women and families experiencing bereavement, wishes and needs assessments, connecting nurses with peer support, and formulating a debriefing plan for staff members [12]. Although guideline development for PBC is difficult due to a lack of empirical evidence and the emotionally burdensome nature of the experience, high-quality bereavement care is critical for women and families following perinatal death [7]. It is urgently necessary to address nurses’ educational needs related to PBC. A prerequisite for this is the development and dissemination of the educational materials related to PBC for Korean nurses.

As the three domains of NAPBS—attitude, importance of policies, and importance of training—were found to be closely related, supporting nurses in terms of attitudes, related policies, and the provision of training or education on PBC may facilitate provision of PBC. Medical institutions and hospitals in Korea should therefore establish clear policies and supportive programs related to PBC for nurses.

Regarding the NAPBS, each of the three subdomains can be considered separately. First, regarding attitudes toward PBC, nurses showed a positive attitude toward giving the parents sufficient time for the bereavement process, but they seemed to hesitate and worry about showing the dying baby to the parents. This is similar to the finding of a previous Korean study [24] that nurses felt conflicted about whether to accept or reject parents’ requests to see their dead baby. However, in a systematic review, Kingdon et al. [25] reported that showing parents their dead baby and giving them the chance to hold the baby could help in the parents’ bereavement process. No guideline or protocol currently exists regarding whether parents can view or photograph their dead baby in Korea; therefore, culturally-specific conversations among health professionals are needed to address this issue. Second, regarding policies, our sample of nurses showed a high level of recognition of the importance of all staff members understanding policies related to PBC, which supports the findings of Chan et al. [26] from three cities in Asia. Third, in relation to the importance of training, nurses placed the highest importance on seeking support when they were emotionally exhausted. This finding is similar to the results of a previous study [27], in which Korean nurses working in the NICU sought social support as their coping mechanism. Furthermore, this supports another Korean study that reported nurses had the strongest demand regarding stress and exhaustion among the palliative nursing education needs [28].

In regard to nurses’ stress related to PBC, the findings of high levels of stress when informing parents of a poor prognosis and coping with PBC in combination with a heavy workload are consistent with those of previous studies in Korea; on nurses caring for dying adults [29-31] and neonates in the NICU [22]. Therefore, administrative efforts are needed in the hospital setting to improve the efficiency of the distribution of nurses’ workload and to provide spaces for parents to mourn when perinatal death occurs. Nurses who will experience PBC could be monitored for their feelings, emotions, and stress related to PBC and counseled at any time before or after PBC. This study’s findings of an association between nurses’ stress and attitudes toward PBC and the perceived importance of training for PBC, can be interpreted as implying that when nurses perceive PBC as a nursing duty for which they are responsible, they easily feel stress and need professional training. Thus when providing PBC education for nurses, we should consider their burden and stress related to PBC and perform an intervention to reduce nurses’ sense of stress or pressure.

Regarding the relationships of general characteristics to the NAPBS, it was found that previous training on PBC was related to a positive attitude toward PBC, consistent with the findings of Chan et al. [9], which emphasizes the importance of nurse training on PBC. Nurses were more aware of the importance of policies when their departments had a clear policy about bereavement care, which is similar to another study in Korea [32], indicating that policies or protocols should be established to improve nurses’ recognition and performance of bereavement care. In this study, there was an unclear relationship between the frequency of PBC and the perceived importance of training for PBC. A possible interpretation may be that as nurses came to have more experiences of PBC, or nurses experienced PBC relatively infrequently, they perceived PBC training as more important or valuable or became increasingly aware of the difficulties and their lack of confidence regarding PBC.

The limitations of this study are as follows. First, participants were selected using convenience sampling, which could interfere with the generalizability of the results to all nurses, especially since variations exist in hospital policies, departmental characteristics, and patient severity. As the Korean version of the NAPBS used in this study was translated and used for the first time, qualitative research on Korean nurses’ attitudes toward PBC, would be beneficial to reexamine the domains of the tool and possibly reconstruct it. Also, nurses’ stress was measured using a tool developed for nurses working in the NICU and may be limited in fully reflecting nurses’ stress for perinatal death outside of the NICU, such as in the delivery room. Thus, there is a need to develop a tool capable of sensitively measuring nurses’ stress related to PBC.

In conclusion, nurses’ attitudes toward PBC were higher if they had received training on perinatal death and if relevant policies had been clearly established in their workplace. More positive attitudes toward PBC were associated with higher stress. Therefore, clear policies for PBC should be implemented, and relevant education programs for nurses should be developed. In order to reduce nurses’ stress related to perinatal death, institutional efforts are necessary to prevent nurses from becoming exhausted and to support nurses in the PBC.

## Figures and Tables

**Table 1.** General characteristics of the participants (N=136)

Variable	Categories	Mean±SD or n (%)
Age (year)	Range: 23–55	31.33±6.73
	20–24	11 (8.1)	
	25–29	61 (44.9)	
	30–34	29 (21.3)	
	≥35	35 (25.7)	
Sex	Female	136 (100)	
Religion	Protestant	29 (21.3)	
	Catholic	16 (11.8)	
	Buddhist	12 (8.8)	
	None	79 (58.1)	
Marital status	Unmarried	88 (64.7)	
	Married	48 (35.3)	
Having children	Yes	27 (19.9)	
	No	7 (5.1)	
Education	Associate degree	12 (8.8)	
	Bachelor degree	105 (77.2)	
	Master degree or more	19 (14.0)	
Bereavement experience in the past year	Yes	22 (16.2)	
	No	113 (83.1)	
Received education about perinatal bereavement care	Yes	10 (7.4)	
	No	124 (91.2)	
	No response	2 (1.5)	
Have a clear policy for the management of bereavement in the workplace	Yes	61 (44.9)	
	No	69 (50.7)	
	No response	6 (4.4)	
Work role	Staff nurse	112 (82.4)	
	Charge nurse	20 (14.7)	
	Advanced practice nurse	4 (2.9)	
Current working unit	Delivery room	64 (47.1)	
	NICU	60 (44.1)	
	Nursery	4 (2.9)	
	Maternity unit	8 (5.9)	
Clinical career (month)	Range: 12–396	100.16±81.93
	≤24	19 (14.0)	
	25–48	20 (14.7)	
	49–72	29 (21.3)	
	≥73	68 (50.0)	
Clinical career in the maternity or neonatal unit (month)	Range: 5–319	73.44±58.39
	≤24	27 (19.9)	
	25–48	28 (20.6)	
	49–72	28 (20.6)	
	≥73	52 (39.0)	
Frequency of facing circumstances involving bereaved parents (time)	Less than once a year	19 (14.0)
	Once a year	36 (26.5)	
	Once every 3 months	46 (33.8)	
	Once a month	29 (21.3)	
	Once a week	3 (2.2)	
	Irregular	3 (2.2)	
Total number of experiences of perinatal death (time)	< 5	45 (33.1)	
	5–9	28 (20.6)	
	10–14	20 (14.7)	
	≥15	43 (31.6)	

NICU: Neonatal intensive care unit.

**Table 2.** Levels of Nurses’ Attitudes towards Perinatal Bereavement Support (N=136)

Contents	Mean±SD
Attitude toward perinatal bereavement care	52.59±4.94
I agree that parents should be given time to grieve.	4.54±0.54
I agree that parents should be treated with respect and dignity.	4.51±0.56
All those who care for and support bereaved parents should have access to support for themselves.	4.36±0.53
I agree that parents should be supported in making their own decisions about what happens to them.	4.34±0.57
All those involved in the care of bereaved parents should be well informed.	4.26±0.79
I agree that a private room should be arranged for a woman who is suffering from intrauterine death.	4.12±0.81
I respect bereaved parents’ feelings and needs.	4.12±0.60
I will communicate with parents in a clear, sensitive, and honest manner.	4.07±0.62
I believe that a grief counseling program can provide psychological support to the bereaved couple.	3.98±0.68
I agree that parent support groups can provide support to parents with similar experiences.	3.93±0.75
I am confident in providing perinatal bereavement support to bereaved couples.	3.68±0.66
I believe that a photograph and footprints can assist parents in working through their grief.	3.35±1.33
I will encourage the bereaved couples to view and hold their baby’s body.	3.32±1.25
Perceived importance of policy to perinatal bereavement care	15.86±2.03
The policy should be understood by all staff involved.	4.24±0.57
The policy should be clearly informed to all staff involved.	4.21±0.62
Nurses should feel assured that they are working within an operational policy which is adequate and appropriate.	4.05±0.62
The unit should have a clear policy for the management of bereavement.	3.37±0.98
Perceived importance of training for perinatal bereavement care	34.72±3.70
Seeking support when feeling burnout.	4.58±0.58
Nurses involved in the care and support of bereaved parents need to be equipped with relevant knowledge, skills, and understanding.	4.46±0.51
Sharing the experience with colleagues and working as a team.	4.41±0.55
Nurses need to feel confident that they are providing adequate and appropriate care.	4.37±0.54
Nurses need to know that they have a limitation when providing perinatal bereavement care.	4.30±0.58
Nurses need opportunities to express their own feelings and needs.	4.23±0.69
Participating in bereavement care.	4.21±0.65
Joining training program on bereavement care.	4.17±0.67

**Table 3.** Levels of nurses’ stress related to perinatal bereavement care (N=136)

Contents	Mean±SD
Lack of knowledge	19.80±2.87
When you cannot give emotional support to bereaved parents due to a lack of communication skills	4.14±0.72
When an emergency situation cannot be handled quickly	4.08±0.96
When it is difficult to give systematic care to a dying baby	3.93±0.80
When knowledge of terminal care is not sufficient	3.88±0.80
When the treatment and nursing care of a dying baby is not timely	3.77±0.91
Lack of an appropriate physical and structural environment	39.25±5.73
When talking to bereaved parents about the poor prognosis of a baby	4.25±0.71
When administrative work is delayed after a death of baby	4.03±0.90
When turning away from the sadness of bereaved parents	4.01±0.83
When there is no guideline or policy for bereavement care	3.98±0.84
When bereaved parents do not have enough time spent with their dying baby	3.97±0.80
When it is difficult to give a private room to bereaved parents	3.95±0.83
When having to explain the administrative work of funeral procedures after death	3.82±1.07
When parents want ongoing life-sustaining treatment, even if the baby’s condition is hopeless	3.80±1.02
When bereaved parents do not accept their baby’s death after a doctor declares it	3.76±1.07
When parents do not make any decision about a dying baby’s care plan	3.68±0.92
Difficulties related to end-of-life care practice	23.46±3.72
When you have to care for a dying baby with a heavy workload	4.25±0.83
When carrying out post-mortem treatment directly	4.07±1.01
When caring for dying babies frequently	4.04±0.85
When a terminal baby’s care brings a work overload	3.90±1.06
When nursing a dying baby is physically exhausting due to excessive physical labor	3.79±1.04
When bereaved parents have a lot of requirements when a baby is about to die	3.41±1.16
Emotional stress	39.25±5.73
When a long-term nursing baby dies	4.06±0.94
When you think you would experience a dying baby again	3.97±1.05
When caring for another baby before grieving for a baby	3.82±0.99
When your feelings become dull as you experience repeated death	3.80±0.99
When you need to perform temporary symptomatic nursing care and not care for a therapeutic purpose	3.63±0.86
When trying to forget a dying baby, but not finding it easy	3.52±0.99
When you cannot talk about your feelings after experiencing the death of a baby	3.49±0.94
When a baby’s death is felt to result from the nurse’s own failure	3.37±1.26
Total	112.16±13.46

**Table 4.** Associations of nurses' attitudes towards perinatal bereavement domains and stress related to perinatal bereavement care (PBC) according to general characteristics (N=136)

Characteristics	Categories	n	Attitude toward PBC		Perceived importance of policy on PBC		Perceived importance of training for PBC		Nurses’ stress related PBC	
			Mean±SD	r or *t* or *F (p)*	Mean±SD	r or *t* or *F (p)*	Mean±SD	r or *t* or *F (p)*	Mean±SD	r or *t* or *F (p)*
Age (year)				.13^‡^		.02^‡^		–.04^‡^		.01^‡^
				(.064)		(.403)		(.323)		(.452)
Religion	Have	56	53.43±5.30	1.67	15.75±2.24	–0.53	35.30±3.91	1.55	113.38±14.34	0.88
	Not have	80	52.00±4.61	(.097)	15.93±1.88	(.599)	34.31±3.51	(.124)	111.31±12.83	(.381)
Marital status	Unmarried	88	52.07±4.48	1.67	15.86±2.11	–0.03	34.69±3.48	0.12	111.65±12.91	0.60
	Married	48	53.54±5.61	(.097)	15.85±1.90	(.979)	34.77±4.10	(.907)	113.10±14.50	(.548)
Education	Associate	12	52.50±5.99	0.49	16.08±2.43	0.09	36.25±3.57	1.53	117.08±9.86	0.93
	Bachelor	105	52.41±4.86	(.615)	15.83±2.03	(.917)	34.45±3.71	(.220)	111.52±13.59	(.398)
	Master	19	53.63±4.81		15.89±1.88		35.26±3.60		112.58±14.61	
Bereavement experience in the past year	Yes	22	52.74±4.43	–0.16	15.36±2.54	1.25	34.95±3.42	–0.32	113.05±13.10	–0.34
	No	114	52.56±5.04	(.875)	15.96±1.92	(.212)	34.68±3.76	(.747)	111.99±13.59	(.738)
Received education about perinatal	Yes	10	57.40±3.34	–3.38	16.90±2.47	–1.69	36.70±3.13	–1.78	109.30±15.20	0.64
bereavement care	No	124	52.14±4.82	(.001)	15.79±1.96	(.094)	34.54±3.72	(.078)	112.14±13.30	(.522)
Had a clear policy for the management of bereavement in my working hospital	Yes	61	53.14±4.99	–1.53	16.52±1.79	–4.23	35.26±3.60	–1.80	112.46±12.56	–0.54
	No	69	51.83±4.79	(.129)	15.13±1.95	(<.001)	34.10±3.74	(.074)	111.14±14.35	(.587)
Work role	Staff nurse	112	52.33±4.64	2.07	15.71±2.06	1.95	34.58±3.53	1.26	111.50±12.72	1.52
	Advanced practice nurse	4	57.25±4.35	(.130)	17.25±1.50	(.148)	37.50±3.70	(.288)	122.75±10.53	(.222)
	Charge nurse	20	53.10±6.28		16.40±1.82		34.95±4.52		113.75±17.23	
Current working unit	Delivery room^a^	64	51.14±5.21	5.58	15.70±2.19	0.85	34.28±4.01	0.84	109.98±15.69	1.19
	NICU^b^	60	54.14±3.89	(.001)	15.88±1.80	(.470)	35.15±3.30	(.474)	113.96±11.20	(.318)
	Nursery	4	56.50±5.07	(b>a)^†^	17.25±2.75		36.25±5.68		118.00±7.75	
	Maternity unit	2	50.62±5.83		16.25±2.05		34.25±2.71		113.13±10.30	
Clinical career (month)				.12^‡^		.04^‡^		–.03^‡^		–.03^‡^
				(.089)		(.314)		(.345)		(.356)
Clinical career in maternity or neonatal (month)				.22^‡^		.19^‡^		.16^‡^		.01^‡^
				(.005)		(.015)		(.029)		(.128)
Frequency of caring for grieving parents	Less than once a year	19	52.91±4.02	1.52	15.84±2.46	0.41	34.47±3.01	0.41	114.67±2.61	1.21
	Once a year	36	54.21±4.52	(.189)	15.79±1.91	(.843)	35.27±3.54	(.839)	112.27±11.97	(.307)
	Once a quarter of a year	46	52.61±5.76		15.90±1.70		34.73±4.00		111.37±12.64	
	Once a month	29	50.90±4.73		15.57±2.38		34.50±3.26		112.75±14.85	
	Once a week	3	51.00±2.83		16.00±1.41		36.50±2.12		129.00±4.24	
	Irregular	3	51.84±4.34		16.54±2.11		33.84±5.16		106.84±18.47	
Total number of experienced perinatal death (time)	< 5^a^	45	53.79±4.14	1.96	16.18±2.08	4.47	35.53±3.21	4.15	113.93±11.80	1.43
	5–9^b^	28	50.96±4.48	(.122)	15.11±1.77	(.005)	32.75±4.24	(.008)	107.86±14.97	(.236)
	10–14^c^	20	52.26±5.55		14.95±2.14		34.30±3.29	(a,d>b)^†^	111.10±12.98	
	≥15^d^	43	52.55±5.50		16.44±1.86		35.35±3.58		113.60±14.07	

NICU: Neonatal intensive care unit.

† Scheffé test,

‡ Pearson correlation coefficients.

**Table 5.** Relationships among nurses' attitudes towards perinatal bereavement (NABPBS) and stress related to perinatal bereavement care (PBC)

Variable	NAPBS, r (*p*)			Stress related to PBC, r (*p*)		
	Attitude toward PBC	Perceived importance of policy on PBC	Perceived importance of training for PBC	Lack of knowledge	Lack of physical and structural environment	Difficulties related to end-of-life care practice
NAPBS						
Attitude toward PBC	1					
Perceived importance of policy on PBC	.40 (<.001)	1				
			
Perceived importance of training for PBC	.61 (<.001)	.52 (<.001)	1			
		
Stress related to PBC						
Lack of knowledge	.29 (.001)	.06 (.460)	.29 (.001)	1		
Lack of physical and structural environment	.24 (.004)	.05 (.588)	.33 (<.001)	.38 (<.001)	1	

Difficulties related to end-of life care practice	.14 (.110)	.05 (.535)	.27 (.002)	.39 (<.001)	.49 (<.001)	1
Emotional stress	.24 (.005)	.019 (.824)	.27 (.001)	.50 (<.001)	.46 (<.001)	.52 (<.001)
Total scores of stress related PBC	.29 (<.001)	.06 (.261)	.38 (<.001)			
